# Reforming Medical Education in China: A Traditional Chinese Medicine Perspective

**DOI:** 10.15694/mep.2017.000032

**Published:** 2017-02-17

**Authors:** Jingyi Fan, Miao Hua, Hongmei Dong, Renslow Sherer

**Affiliations:** 1 Zhongnan Hospital of Wuhan University; 2 University of Chicago

**Keywords:** Medical education, traditional Chinese medicine, medical reform

## Abstract

This article was migrated. The article was marked as recommended.

**Introduction**: While the Chinese medical education system is undergoing comprehensive reform, traditional forms of Chinese medicines (TCM) continue to be a unique and indispensable part of health care system. However, few studies have explored how various forms of TCM are incorporated with biomedicine in clinical practice.

**Purpose**: To explore clinicians’ professional and extraprofessional experience with TCM and to assess whether their medical education has prepared them for clinical work that requires drawing on knowledge of TCM.

**Methods**: Surveys were distributed in 2013 to 18 clinicians, 33 undergraduates and 60 post-graduate students. The survey combined forced-choice and open-ended questions assessing personal and professional experiences with TCM. Mixed qualitative and quantitative methods were used to investigate trends in open-ended survey responses.

**Results**: The majority of clinicians (89%), post-graduate students (60%) and undergraduate students (67%) have personally used TCM treatments. The vast majority of all three groups indicated that they would continue to recommend TCM to patients. Respondents expressed an overall positive attitude towards their extraprofessional experience with TCM whereas their professional experience with TCM was mixed.

**Conclusion**: The extraprofessional and professional experiences of clinicians and students with various types of TCM for a diverse array of indications reveal the sustained clinical presence of TCM. The survey reveals the importance of more training in applying TCM, especially in a clinical setting, and imminent hurdles that must be overcome in implementing clinical training reforms.

## Background

The Chinese medical education system has been undergoing state-mandated reform since the turn of the millennium, and with greater impetus since 2008 ( Yang et al. 2012). These reforms impinge on a medical education and health care system in which traditional medicine and biomedicine are jointly administered and delivered at every level. The important place of plurality in the Chinese health care system is indicated by the State Council’s 2015 directives on health care reform, which stipulates that 15% public hospital the Chinese state will subsidize by 2020 are reserved for traditional medicines (including traditional Chinese and other minority medicines) ( Office of the State Council 2015). Studies have noted the uniqueness of this dual-track system ( Hesketh et al. 1997; Lam et al. 206). However, few have systematically studied how doctors trained in biomedicine regularly employ traditional medical therapeutics in clinical practice.

Traditional Chinese medicine (TCM) has thrived over the past 60 years under state administration, with hospital systems, medical universities, and professional status in parallel with those of “Western medicine.” Teaching TCM in schools of Western medical (or biomedical) schools in China can be compared with the treatment of complementary and alternative medicine (CAM) in U.S. medical schools, but it is a comparison that reveals more contrasts than similarities. In 1997, the American Medical Association proposed incorporating elective CAM curriculum medical schools, leaving individual schools free to decide the extent and makeup of course requirements ( Wetzel et al. 1998). In China, the Ministry of Education stipulates that 5-year medical programs in China provide training in traditional medicine as part of mandatory coursework. TCM, more so than CAM in the U.S., is considered an integral facet of a biomedical education. A survey conducted in China over ten years ago shows that students in biomedicine universities receive two semesters of training in traditional Chinese medicine, amounting to over 200 hours of didactic experience ( Lew et al. 2004). How the distribution of these hours vary across institutions and how such coursework has been received remains under-investigated, part of the gap that this study strives to fill in.

At Zhongnan Hospital, a tertiary teaching hospital affiliated with Wuhan University School of Medicine (WUSM) where we conducted our study, the Department of Integrated Chinese and Western Medicine is staffed mostly by clinicians trained in TCM. Medical students can elect to rotate at this department, but there is no mandatory TCM rotation. In 2013, we surveyed clinicians and students at Zhongnan Hospital/WUSM from different specialties to evaluate their clinical exposure and extraprofessional experience with TCM.

The purpose of this preliminary study is to investigate two key questions. First, it attempts to characterize with greater detail the extent of professional and extraprofessional experience with traditional Chinese medicine within different cohorts of medical practitioners who identify predominantly as biomedical practitioners. Up until now, much of the research on TCM has been and can be better integrated with “Western medicine” has focused on basic sciences and translational research ( Lu & Wu 2008; Lu et al. 2012). Less attention has been granted to ongoing and continuous integration of traditional Chinese and Western medicines at the bedside. Second, as state-led medical reform expands to encompass residency training for physicians of all specialties, how medical schools and teaching hospitals should take into consideration already existing interconnections between biomedicine and TCM at the bedside needs to be countenanced anew.

## Method

### Survey Instrument

Copies of a Chinese-language survey were distributed over July-August, 2013. Post-graduate and undergraduates students were administered one version, clinicians were administered another version. The survey contained three sections: “extraprofessional experience,” “professional experience” and “educational experience.” Respondents were asked to provide a yes/no response whether they have had (1) personal experience with TCM or use among family/friends, (2) clinical experience with TCM, and (3) formal instructions on TCM. Those who indicated that they had experience with TCM in any of the three categories were also asked to give a “positive” or “negative” evaluation of their experience. Forced-choice questions asked respondents to indicate types of patients they thought were best suited for TCM therapies, and the types of therapies they would recommend. Respondents had the option of providing detailed descriptions of their experience with TCM in the format of open-ended questions. The “student” version of the survey differed from the “clinician” version in that the former asked respondents whether there would like to see more TCM incorporated into their training, and their preferred setting, while the latter were only asked to evaluate the effectiveness of the training they did receive.

### Participants

A convenience sample was taken of clinicians and students. A roster of 18 clinicians became sample subjects, their names available to the surveyors because all of them have travelled on visiting fellowships to the United States. Undergraduate students and post-graduate students were approached at separate extracurricular events. These events had no obvious connection with TCM. Thirty-three undergraduates in years 4 and 5 of their medical school training and 60 post-graduate students agreed to participate in this study and stayed to fill in the survey. 1 ^st^-3 ^rd^ year undergraduates were excluded from this study for their relative lack of clinical experience.

### Analyses

Participants’ answers to the open-ended questions were first coded by one of the authors independently to generate main categories and subcategories. Then another author coded the same data again and compared with the first coder’s results. The coder agreement rate was around 90%. Then the two coders discussed and adjusted their results, arriving at 100% agreement. Participants’ responses to forced-choice items were tallied and percentages were calculated. The same was done with categories that emerged from qualitative analyses. Since many survey questions were open-ended, tallies were made of the number of times categories were spontaneously mentioned, in contrast to the lack of mention of certain terms. This mixed use of qualitative and quantitative techniques allowed a more nuanced description of trends in the open-ended responses.

## Results

### Demographics

The Clinicians (18) were from 12 different departments including oncology, obstetrics and gynecology, endocrinology, cardiology, community medicine, hematology, gastroenterology, nephrology, urology, general surgery, hematology and pediatrics. They represented most of the subspecialties at Zhongnan Hospital with the exception of neurology, dermatology, infectious diseases, rheumatology and other surgical subspecialties including ophthalmology and orthopedics.

60 post-graduate students in their first, second, or third post-graduate years surveyed. All them are graduates of 5-year medical programs doing clinical training at Zhongnan Hospital or other teaching hospitals. 33 undergraduate medical students from WUSM in their fourth and fifth year were surveyed. The total number of participants was 111.

### Extraprofessional Experiences with TCM

The majority of all three groups of subjects had personal experience with TCM treatments. Among those who admitted to personally using traditional Chinese medicine or use among family/friends, all three demographics indicated that it was an overall positive experience when asked to select between a positive/negative binary (see Table 5). A smaller portion of respondents went on to answer open-ended questions asking them to describe the exact nature of the experience and treatment efficacy. Respondents could answer two separate questions per survey on heir extraprofessional experiences, one on their personal experience, one on experiences of family members and friends. These responses were compiled for data analysis. In total, 32 open-ended responses were gathered from clinicians, 45 from post-graduates, and 46 from undergraduates.

We analyzed the responses to the open-ended questions, grouped according to sets of qualitative categories ( Table 1). Experiences using TCM outside the professional setting are regular and diverse, so we counted the types of conditions and treatments spontaneously mentioned. These frequencies are enumerated in the parentheses in Table 1. The most common types of condition falling under a broad category of “internal medicine” include upper respiratory infections, chronic laryngitis, asthma, digestive disorders, heart conditions, cancer, and chronic disorders not otherwise specified. The most common treatments sought out by far are oral medications, including patent Chinese medications (pre-formulated traditional medications in easily ingestible pill-form). Of note, a significant number of responses indicate that various types of therapies, both externally applied and internally ingested, are pursued for chronic use. Experiences of efficacy or adverse reactions were mentioned less frequently, almost entirely absent from the postgraduate demographic. But it is significant that over half of clinicians who did respond to the open-ended questions recall specific instances of improvement due to TCM, and fewer instances of mixed or lack of efficacy. Overall, clinicians’ responses were richer in detail and evince greater knowledge about outcomes than those of the students.

**Table 1.** Qualitative summary of extraprofessional experiences with TCM

	Types of Conditions	Types of Treatments	Efficacy
**Clinicians (32 reports)**	Internal medicine (5), ob-gyn (4), musculoskeletal and rehabilitation (4), insomnia (2), and single instances of miscellaneous conditions including verruca plana, breast nodule, brittle hair, and disposition towards coldness	Oral (herbal) medication (20), *tuina*-massage (10), acupuncture/moxibustion (8), and cupping (1). Therapies were described as one-time course (10) or long term-use (7)	20 reported on efficacy: alleviation of personal complaints (9), relief for family/friends (6), no significant relief for family/friends (4), mixed results for family/friends (1)
**Post-grads (45 reports)**	Internal medicine (10), ENT (6), musculoskeletal & rehabilitation (3), ob-gyn (2) and single instances of miscellaneous conditions including nephrolithiasis, weight loss, and general tonifying	Oral (herbal) medication (20), acupuncture/moxibustion (5), tuina-massage (2), injectable herbal medication (1), and cupping (1). Therapies were described as one-time course (17) or long term-use (4)	2 reported on efficacy, both positive
**Undergrads (46 reports)**	Internal medicine (10), musculoskeletal and rehabilitation (2), ob-gyn (1), and single instances of miscellaneous conditions including facial acne, hemorrhoids, and chronic pain/fatigue.	Oral (herbal) medication (23), acupuncture/moxibustion (3), physical therapy/tuina-massage (2), external application of herbal medicines (2), medicinal wine (1). Therapies were described as one-time course (8) or long term (3).	7 reported on efficacy: alleviation of personal complaint (1), efficacy with personal use was uncertain (1), efficacious for family/friends (4), not efficacious for family/friends (1)

### Professional Experiences with TCM

Survey respondents were also asked to evaluate their clinical experience with TCM. The vast majority of all three groups admit to having prescribed TCM treatments for patients, or having witnessed more senior physicians prescribing TCM ( Table 5: last two columns). There is a clear tilt towards more responses and greater details furnished in the responses to open-ended questions among those with greater clinical experience: clinicians more than post-graduates more than undergraduates. All 17 out of 18 clinicians responded to open-ended questions. Out of post-graduate and under-graduate students, 42 and 13 answered the open-ended questions, respectively.

Professional experiences with TCM that the three groups reported contain some patterns, categorized in Table 2. First, even though not enough reports of indication were gathered to offer any conclusions about the most common diagnoses for which TCM is indicated, physicians from diverse specialties were able to name a diverse set of indications. Second, clinicians as well as students indicate that they or their superiors regularly prescribe patent Chinese medications. This is distinct from “consulting TCM physicians,” which is the second most frequently reported “manner of use,” suggesting that non-TCM physicians are quite comfortable prescribing TCM medications, in particular patent Chinese medicines. The same cannot be said for prescribing herbal formula containing combinations of dried herbs that have not been decocted or otherwise processed, a “manner of use” rarely described ([C]: 0; [PG]: 2; [UG]: 1). One clinician reports consulting TCM physicians to prescribe herbal formula, suggesting that it is not lack of interest but of comfort and expertise on their part. Not all biomedically-trained physicians are equally shy about prescribing herbal formula, as a few students recall their supervisors doing so. Third, where efficacy is mentioned, it is generally positive, but one clinician reported an adverse event.

**Table 2.** Qualitative summary of professional experiences with TCM

	Indications	Manner of use	Efficacy
**Clinicians (17 reports)**	Pancreatitis (1), endometriosis (1), rehabilitation unspecified (1), viral URI (1), post-operative ileus (1)	Prescribe patent Chinese medications (11), consult TCM physicians (7), ask TCM physicians to prescribe herbal formula (1)	7 reported on efficacy: symptomatic improvement (6), adverse reaction (1)
**Post-grads (42 reports)**	Prostatitis (2), renal failure (1), congestive heart failure (1), small bowel obstruction (1), cardiovascular disease (1), cancer (2), unspecified post-operative (2), cervical strain (1)	Prescribe patent Chinese medications (23), consult TCM physicians (9), prescribe herbal formula (2), prescribe injectable Chinese medication (2), acupuncture (1), cupping (1)	1 report of improvement
**Undergrads (13 reports)**	Osteosarcoma (1), hematology-oncology unspecified (2), rehabilitation unspecified (1)	Prescribe patent Chinese medications (7), consult TCM physicians (3), prescribe herbal formula (1)	No report

The surveys also asked if respondents would recommend TCM to patients in the future. Those who answered “yes” to the question selected the types of complaints they would consider for recommending TCM treatments ( Table 3), as well as indicate the types of therapy they would recommend ( Table 4). The vast majority of all three groups expressed that they would continue to recommend TCM to patients. For all three groups, “chronic” was the category of conditions most considered amenable to TCM therapies. In second place was “ob-gyn,” followed by “infertility.” The “other” category was widely selected by clinicians, who described dermatological (5), orthopedic (5), oncological (1), rheumatic (1) conditions as well as “difficult cases” recalcitrant to Western medical methods (1). Post-graduates and undergraduates did not volunteer as diverse an assortment of categories not listed. Notably, all three groups suggested “oncological” as an additional category. Like one of the clinicians, one post-graduate respondent also brought up “difficult cases” that do not respond to “Western medicine” as amenable to TCM treatment.

**Table 3.** Types of patients to recommend TCM

	Chronic	Ob-Gyn	Infertility	Palliative	Pediatric	Acute	Other
**[C] *n=18 (100%)***	17 (94%)	10 (56%)	7 (39%)	4 (22%)	2 (11%)	3 (17%)	13 (71%)
**[PG] *n=54 (90%)***	48 (80%)	20 (33%)	13 (22%)	9 (15%)	5 (8%)	4 (7%)	4 (6%)
**[UG] *n=29 (88%)***	27 (93%)	21 (72%)	14 (45%)	11 (38%)	4 (14%)	1 (3%)	2 (3%)

Herbal medications are by far the most widely recommended type of therapy. After that, acupuncture-moxibustion, cupping, *tuina*-massage, and clinical consultation to TCM clinicians were more or less evenly selected as recommendable methods. The results are summarized in Table 4.

**Table 4.** Types of TCM to recommend patients

	Herbal Medicines	Acupuncture-moxibustion	Tuina-massage	Cupping	Clinical Consultation	Bone-setting	Other
**[C] *n=18 (100%)***	13 (72%)	8 (44%)	10 (56%)	6 (33%)	11 (61%)	2 (11%)	2 (11%)
**[PG] *n=54 (90%)***	38 (70%)	28 (52%)	23 (43%)	21 (50%)	18 (33%)	11 (39%)	0 (0%)
**[UG] *n=29 (88%)***	25 (90%)	18 (62%)	15 (52%)	12 (41%)	13 (45%)	4 (14%)	1 (3%)

Overall, the extraprofessional and professional experiences of clinicians and students with various types of TCM encompass a diverse set of indications suggestive of the sustained clinical presence of TCM. Respondents to the survey express an overall positive attitude towards their extraprofessional experience with TCM. Their professional experience with TCM at the bedside is more split between positive and negative/mixed attitudes. Table 5 summarizes clinicians’ and students’ professional and extraprofessional experiences in using or prescribing TCM and if they had an overall positive experience. Those who did not indicate a “positive” experience would have selected “negative” or chose not to respond.

**Table 5.** Summary of professional and extraprofessional experiences with TCM

	Personal	Positive	Family & Friends	Positive	Clinical	Positive
**[C] *n=18***	16 (89%)	16/16 (100%)	16 (89%)	9/16 (75%)	18 (100%)	10/18 (56%)
**[PG] *n=60***	36 (60%)	23/36 (64%)	45 (76%)	26/45 (58%)	54 (90%)	26/54 (48%)
**[UG] *n=33***	22 (67%)	16/22 (84%)	24 (73%)	14/24 (58%)	17 (53%)	9/17 (53%)

### Training in TCM

The majority of respondents received training in TCM. Opinions are split among all three demographics regarding whether they consider their training to have been effective. Table 9 summarizes respondents’ educational experience with TCM and their extant attitudes toward that education. The student version of the survey also asked participants if they would be interested in further TCM education, and whether they had a preference for a clinical or classroom setting. Again, opinions were split regarding the expansion of TCM training. Many students complained that Chinese medical theory taught in the classroom was challenging and mysterious (n = 19). The vast majority of students indicated that given the opportunity to train further in traditional Chinese medicines, they would prefer a clinical education. The most common rationale students provided for this preference was the importance of “experiential/practical training” (n = 17). Among those who favored both clinical and classroom settings, the explanation given in all responses was “to better integrate theory and practice” (n = 4).

**Table 6.** Education in TCM and attitudes

	Received Training	Training was Helpful	Interest in Further Training	Preferred Setting: Clinical	Preferred Setting: Classroom	Preferred Setting: Both
**[C] *n=18***	15 (83%)	8/15 (53%)	---	---	---	---
**[PG] *n=60***	48 (80%)	24/48 (50%)	28 (47%)	36 (60%)	3 (5%)	7 (12%)
**[UG] *n=33***	31 (94%)	18/31 (58%)	17 (52%)	18 (55%)	3 (17%)	8 (24%)

## Discussion

Results from the surveys suggest that TCM is ubiquitous in the professional and extraprofessional lives of biomedically-trained physicians in China. Previous studies have also shown that physicians frequently encounter TCM the bedside ( Lew et al. 2004; McQuade et al. 2012). We have moreover shown that the majority of clinicians, post-graduate students and undergraduate students have personally used or know of family members and friends who have resorted to TCM treatments. Professional experiences of the survey respondents further indicate that TCM is in widespread use. A small majority of undergraduates have witnessed TCM being used at the bedside. This becomes a large majority with post-graduates, as 90% of respondents had exposure to some form of clinical use of TCM. All clinicians surveyed have recommended TCM to patients. This suggests that clinical familiarity with TCM is cumulatively acquired in the post-graduate years and beyond. The majority of all three cohorts report positive extraprofessional encounters with traditional Chinese medicine, whereas professional attitudes toward TCM are more mixed. Almost all are in agreement that they will continue to recommend TCM therapies to patients.

The survey responses suggest that TCM knowledge is acquired experientially. This can be inferred in several ways. Firstly, there are overlaps between the professional and extraprofessional experiences and attitudes of clinicians and students. Oral medications are the class of therapies with which clinicians and students have the most professional and extraprofessional experience. Acupuncture-moxibustion and *tuina*-massage also rank high in both the extraprofessional experiences and as therapies to recommend patients ( Table 4). The three groups of respondents uniformly consider “chronic conditions” to be most suited for TCM intervention ( Table 3). These patterns corroborate commonly held assumption that traditional Chinese medicine is most suited for chronic conditions, less so for acute-onset disorders ( Karchmer 2015). Whether this popular perception is reinforced by clinical reality is unclear, as all three groups report more instances of taking one-time doses of Chinese drugs than chronic ingestion in their extraprofessional experience.

The second pattern that suggests an experiential bias in TCM knowledge can be found in the cross-sectional picture of how different generational cohorts report their exposure to TCM. There is an incline in exposure to TCM from undergraduate medical students to post-graduates and clinicians. The gradual acquisition of bedside experience is consistent with a general lack of early clinical training among Chinese medical students ( Sun 2006). However, even reports of post-graduate students who admit to having witnessed TCM in training are lacking in details about efficacy. Accounts of efficacy, including instances of adverse reaction, were mostly generated by clinicians. The discrepancy implies that clinicians become aware of TCM efficacy, drug interactions and adverse reactions from individual experience. Given that a formal TCM education is mandatory, it is surprising that post-graduate students evince little specific understanding of the efficacy of TCM therapies.

Furthermore, it appears that much of the use of TCM may occur without formal input from TCM physicians. “Consulting a TCM physician” is the second most frequently selected clinical recommendation among clinicians, but only the fourth and sixth most selected recommendation by undergraduates and post-graduates, respectively ( Table 4). This along with the fact that only 53% of undergraduates report witnessing TCM in their clinical experiences suggests that physicians-in-training are not regularly provided opportunities to work with TCM physicians or given clinically specific instructions on how to prescribe TCM medications.

Among all three cohorts, prescribing patent Chinese medication is reported as a common practice for biomedically-trained clinicians (see Table 2). Prescribing formula of loose herbs in combination is much less frequently considered an option. However, the distinction between herbal patent and formula medications is not made when students and clinician report on their personal experience, with the most frequently recorded “Type of Treatment” being the generic category of “oral (herbal) medications” (see Table 1), suggesting that there is implicit knowledge that the two types of medications overlap in indication and mechanism of action. Yet, the line between patent and formula medications is clearly drawn in reports of professional experience. McQuade et al. (2012) also found that use of herbal medications among oncology patients in Shanghai was exceptionally high. 90% of oncologists also admit to having prescribed herbal medications. However, the authors lumped together herbal formula, oral patent medications, and injectable herbal medications, so it is unclear which types of agent clinicians were prescribing. Our qualitative data suggests that biomedical clinicians are less likely to prescribe unprocessed herbs in formula and more comfortable prescribing pre-formulated patent medications.

The importance of more training in actually applying TCM is directly reflected in the surveys. Only a little over half of physicians and students surveyed consider the training they have received to be effective ( Table 6). This may in part reflect the current state TCM education in biomedical universities, which concentrates mainly on Chinese medical theory delivered through didactic formats in the classroom. While only around half of the students evince interest in further TCM training, a clinical education is widely preferred.

Limitations of this preliminary study include lack of equally high response rates for all of the questions administered. Many of the surveys were answered incompletely, so response rates differed from question to question. While the open-ended responses revealed the greatest detail about clinical decision-making and exposure to TCM, the response rate to these questions were low among students. The results of the qualitative analysis are thus skewed by selection bias towards those who have had more experience with TCM. While many of the clinicians’ responses to the open-ended questions were rich in detail, the number of subjects sampled was inadequate to make generalizable conclusions. The sample selection was based on convenience and therefore may not be representative of physicians working in similar patient care settings.

## Conclusion

Medical education in China is in the midst of systemic reform. As of 2015, all 5-year medical graduates must undergo 3 years of residency training in the so-called “5+3” standard to meet the minimum requirement to practice as physicians ( NHFPC 2014). This state-led effort to expand clinical training converges with medical schools pursuing reforms in bringing multi-specialty clinical education into earlier stages of biomedical training ( Zhang et al. 2013). Yet, despite China’s unique dual-track system of “TCM” and “Western medicine,” little effort has been made in assessing how biomedical physicians have received their mandatory training in TCM. This study shows that a sizable percentage of clinicians at a large multidisciplinary Chinese teaching hospital regularly employ TCM for their patients. Most have even approvingly utilized TCM personally. However, almost half of clinicians and medical students surveyed consider their mandatory coursework to have been less than helpful. Knowledge of how to apply TCM has formed unsystematically, based on common assumptions, without differentiation between drug ingredients and classes, and often without consulting TCM physicians. Given that TCM will continue to play an important role in most Chinese clinicians’ therapeutic arsenals, more targeted clinical training in TCM is warranted in the future. Integrating Chinese medical and biomedical education at the bedside, however, harbors numerous challenges that will require greater collaboration between TCM and Western medicine physicians to overcome. This moment of clinical education reform is an important time to assess the current limits and future directions of applying traditional Chinese medicine at the bedside.

## Take Home Messages

• Chinese physicians and clinical students have extensive personal and professional experiences in Traditional Chinese Medicine.
• Most physicians have prescribed herbal medications in the form of patent medications for their patients.
• Clinical training in TCM is disproportionately absent when held up against how widely it is recommended to patients.
• Clinicians of biomedicine have limited practical understanding of TCM, as their training has formed unsystematically after undergraduate-level didactic courses that almost half of survey respondents consider less than helpful.
• During this era of medical education reform in which an unprecedented effort is being exerted statewide to expand multidisciplinary clinical education, reforming TCM education at the bedside can begin with incorporating TCM-trained physicians in the clinical instruction of biomedical students.

## Notes On Contributors

Jingyi Fan, M.D. is an associate professor in Pediatric Department and vice director of the Teaching Office at Zhongnan Hospital of Wuhan University

Miao J. Hua is a doctoral student in anthropology at the University of Chicago and MD candidate at Rosalind Franklin University in Chicago.

Hongmei Dong, Ph.D. is senior research technologist at the Department of Medicine at the University of Chicago.

Renslow Sherer, M.D. is a Professor in the Department of Medicine, Section of Infectious Diseases and Global Health, at the University of Chicago.
