# Supplementary material for: Reforming Medical Education in China: A Traditional Chinese Medicine Perspective
Source: MedEdPublish (2016). 2017 Feb 17;6:32. [Version 1] doi: 10.15694/mep.2017.000032 (PMC10885235; doi:10.15694/mep.2017.000032)
Supplement: Peer review history

# Reviewer report: Reviewer response for version 1

**DOI:** 10.21956/mep.19409.r28482

**Published:** 2017-02-19

**Authors:** Richard Hays

**Affiliations:** 1 James Cook University

This review has been migrated. The reviewer awarded 3 stars out of 5 I also found this to be a very interesting article. My experience in China has shown me just how important TCM is in that country (and throughout much of east Asia), as the population use it widely and so 'western-trained' doctors need to know more about it. It is, however, difficult to extrapolate beyond that region to other nations where western medicine is more dominant and TCM is just one of many alternative approaches. I also have some concerns about the methods. While the results 'feel' about right, a convenience sample is an unusual approach to seeking representative opinions and the samples were small, constraining interpretation. More evidence is needed to support combining TCM and 'western medicine' approaches before medical education is reformed in this direction.

Reviewer Expertise:

NA

No decision status is available

# Reviewer report: Reviewer response for version 1

**DOI:** 10.21956/mep.19409.r28480

**Published:** 2017-02-17

**Authors:** P Ravi Shankar

**Affiliations:** 1 American International Medical University

This review has been migrated. The reviewer awarded 4 stars out of 5 I enjoyed reading this well-written article. The authors have comprehensively described various aspects of education in traditional Chinese medicine (TCM) for biomedical students of western medicine in China. The limitations have also been well described. I have an interest in integrative medicine and have published a few studies in this area. Practitioners of modern western medicine should have an understanding of complementary medicine for a variety of reasons including possible interactions between western medicines and any traditional medicine the patient may be taking. The authors could have added a sentence describing some of the less common TCM remedies. At a few places in the manuscript, there are a few missing words which can be looked into and corrected.

Reviewer Expertise:

NA

No decision status is available

# Reviewer report: Reviewer response for version 1

**DOI:** 10.21956/mep.19409.r28481

**Published:** 2017-02-17

**Authors:** Trevor Gibbs

**Affiliations:** 1 AMEE

This review has been migrated. The reviewer awarded 3 stars out of 5 Being involved quite extensively in healthcare in China meant that I was interested in reading this paper. However, from my personal observations and enquiries with my clinical colleagues from China, I am not really surprised by the findings. Given that the vast majority of the population in China experience TCM from birth, given the edict issued that governs the necessity to maintain the TCM Schools of Medicine and the present integration of TCM into Western-style medical schools, I am not at all surprised by the findings. I would agree that there is a relative lack of training in TCM when one considers the wider spectrum of clinical training , but I would prefer to follow that observation up with a piece of research that asks why this is so .I feel that is not appropriate to compare the integration of TCM in China's schools with that experienced within the US with complementary therapies- they are based upon two different purposes.I felt that the questionnaire was somewhat narrow in its approach and really needed expansion into a more qualitative coverage looking into reasons rather than the binary yes or no Given the title of the paper I wondered if a more critical evaluation of whether TCM is a key reforming activity in China is more appropriate .I am sure that this debate will continue and will look forward to see how it develops

Reviewer Expertise:

NA

No decision status is available
